# Postoperative Complications and Outcome After Emergency Laparotomy: A Retrospective Study

**DOI:** 10.1007/s00268-022-06783-8

**Published:** 2022-10-16

**Authors:** Aura T. Ylimartimo, Juho Nurkkala, Marjo Koskela, Sanna Lahtinen, Timo Kaakinen, Merja Vakkala, Siiri Hietanen, Janne Liisanantti

**Affiliations:** 1 Research Group of Surgery, Anesthesiology and Intensive Care Medicine, Medical Research Center of Oulu, Oulu, Finland; 2 Department of Surgery, Oulu University Hospital, P.O. Box 21, 90029 OYS Oulu, Finland; 3 Department of Anesthesiology, Oulu University Hospital, Oulu, Finland

## Abstract

**Background:**

Emergency laparotomy (EL) is a common urgent surgical procedure with high risk for postoperative complications. Complications impair the prognosis and prolong the hospital stay. This study explored the incidence and distribution of complications and their impact on short-term mortality after EL.

**Methods:**

This was a retrospective single-center register-based cohort study of 674 adults undergoing midline EL between May 2015 and December 2017. The primary outcome was operation-related or medical complication after EL. The secondary outcome was mortality in 90-day follow-up. Multivariate logistic regression analyses were used to identify independent risk factors for complications.

**Results:**

A total of 389 (58%) patients developed complications after EL, including 215 (32%) patients with operation-related complications and 361 (54%) patients with medical complications. Most of the complications were Clavien-Dindo classification type 4b (28%) and type 2 (22%). Operation-related complications occurred later compared to medical complications. Respiratory complications were the most common medical complications, and infections were the most common operation-related complications. The 30- and 90-day mortalities were higher in both the medical (17.2%, 26.2%) and operation-related complication groups (13.5%, 24.2%) compared to patients without complications (10.5% and 4.8%, 14.8% and 8.0%). Low albumin, high surgical urgency, excessive alcohol consumption and medical complications were associated with operation-related complications. Older age, high ASA class and operation-related complications were associated with medical complications.

**Conclusions:**

This study demonstrated that EL is associated with a high risk of complications and poor short-term outcome. Complications impair the prognosis regardless of which kind of EL is in question.

## Introduction

Emergency laparotomy (EL) is a high-risk operation; more than a half of the patients are afflicted with complications [1]. Thirty to 50% of EL patients present with systemic inflammatory response syndrome, sepsis and septic shock, and mortality remains high [2–9]. Most patients undergoing EL are elderly with comorbidities; for example, malignancies are present in approximately 10% of the patients [2, 10, 11].

Postoperative complications and other adverse events following EL prolong hospital stay and impair the outcome [10, 12]. Compared with elective operations, EL patients are up to five times more likely to die within 30 days after their operation [12]. Severe septic shock, multi-organ failure, cardiopulmonary incidents, surgical complications and malignant diseases are the most typical causes of death after EL [2]. Accordingly, among elderly the functional outcomes and return to independency after EL are poor [13].

Although emergency general surgical admissions comprise the largest group of all surgical admissions, several studies have shown a variation in the delivery of evidence-based care and outcomes [1, 14–21]. EL patients have been a relatively overlooked group [22], but studies in the past few years show improvement in outcomes using enhanced recovery protocols among this high-risk patient group [23–28]. The Enhanced Recovery After Surgery (ERAS) Society published guidelines for EL patients in 2021 and suggest this protocol should be routinely applied to the care of EL patients [29].

Although ELs carry a significant rate of complications and high mortality, studies are lacking focusing on the medical complications and time of onset of the observed complications. In the present study, we aim to explore the rate of both medical and operation-related complications and their impact on the short-term mortality after EL. Our hypothesis is that patient-related factors including age and comorbidities play a major role in the incidence of complications; furthermore, we hypothesized that recorded complications have a major impact on mortality.

## Methods

This retrospective register-based cohort study was conducted in Oulu University Hospital, Oulu, Finland. The study protocol was approved by the hospital administration (reference number 66/2018). According to the local protocol, no statement from the Ethics Committee was required because of the retrospective study design.

### Patients

All the patients who had undergone a midline EL between May 2015 and December 2017 were reviewed from the hospital’s discharge records, and the eligible patients were included in the study. The exclusion criteria were age < 18 years, urgent or emergency cholecystectomy or appendectomy, or emergency or urgent laparotomy due to a gynecological cause, leaving a total of 674 EL patients.

### Data extraction

The data were obtained from electronic medical records, anesthesia charts and operation charts. The following data were collected: age, sex, diagnosis, type and duration of the operation, complication type, intensive care unit (ICU) length of stay (LOS) and hospital LOS. The urgency of the operation was classified as follows: immediate operation performed within 3 h after the decision to operate, very urgent operation performed within 3–8 h after the decision to operate and urgent operation performed within 8–24 h after the decision to operate. The severity of the underlying comorbidities was assessed using the Charlson Comorbidity Index (CCI) [30]. The American Society of Anesthesiologists classification (ASA) was used to estimate the patient's preoperative risk [31]. Preoperative levels of albumin, leukocytes, platelets, hemoglobin and C-reactive protein (CRP) were obtained from the patients’ medical records.

### Complications

Postoperative complications recorded by the treating physicians were obtained from the medical records. The analysis included all postoperative complications during the hospital stay. The postoperative complications were classified as operation-related or medical and as minor or major complications. According to the Clavien-Dindo classification, classes I–II were regarded as minor complications and classes III–V as major complications [30]. Operation-related complications included surgical site infection, fascial rupture, bleeding, seroma, anastomotic leakage, strangulation or herniation, and the need for reoperation during the same LOS. Medical complications included pneumonia, respiratory dysfunction, pulmonary embolism, sepsis, acute kidney dysfunction, acute liver dysfunction, stroke, asystole and resuscitation, heart failure and atrial fibrillation. The complications were categorized following the protocol presented in our previous study [32]. The time of onset of complications was determined within the accuracy of 1 day. Complications were categorized as early complications, i.e., the onset within 1–4 days after the operation, or as late complications, i.e., the onset > 4 days after the operation. The times of deaths were retrieved from the hospital's medical records to assess the in-hospital, 30- and 90-day mortality rates.

### Statistical analysis

Due to the retrospective study design, we did not perform a power calculation to assess the sample size. Statistical analyses were performed with IBM SPSS statistics 27 software (IBM SPSS Statistics for Windows, Version 27.0, Armonk, NY, USA). Categorical data are presented as numbers (n) and percentages (%). Continuous variables are expressed as medians and 25th to 75th percentiles [25th–75th]. Comparisons were performed with Pearson's chi-square for categorical data and the non-parametric Mann-Whitney *U* test for continuous data. Two-tailed *p* values <0.05 were considered statistically significant. Logistic regression analysis was performed to calculate odds ratios (OR) for surgical and medical complications. Age and categorical variables with univariate significance <0.05 were included in the model one by one using the enter method. Variables with multivariate significance <0.05 were kept in the model as well as those with significant impact on the log-likelyhood function.

## Results

Complications were recorded after EL in 389 (58%) patients. Operation-related complications were recorded after an EL in 215 (31.9%) patients and medical complications in 361 (53.6%) patients. One hundred eighty-six (27.6%) patients had both a medical and an operation-related complication. Wound infections and intra-abdominal abscesses were the most common operation-related complications. One hundred twenty-nine (19.1%) patients required reoperation. Most of the operation-related complications, except bleedings, were classified as late complications. Respiratory dysfunction, pneumonia and septicemia were the most common medical complications. Medical complications were often early with exception of neurological complications such as transient ischemic attack (TIA). Most of the complications were Clavien-Dindo classification type 4b (28%) and type 2 (22%) (Tables 1 and 2).

**Table 1:** Postoperative complications of 674 patients after emergency laparotomy

Complication	Number of patients with complication	Time of onset of the complication (days)	Number of late complications
Operation-related complications	215 (31.9)	7 [3–11]	
Fascial rupture	34 (5.0)	7.5 [5–10]	27 (79.4)
Wound infection	110 (16.3)	5 [2–9]	63 (57.3)
Superficial	37		
Deep	73		
Bleeding	49 (7.3)	1 [1–9]	17 (34.7)
Seroma	20 (3.0)	6.5 [3–8]	13 (65.0)
Anastomotic leakage	41 (6.1)	8 [4–11]	27 (65.9)
Strangulation/herniation	4 (0.6)	6 [2–15]	2 (50.0)
Intra-abdominal abscess	95 (14.1)	9 [5.5–15]	75 (78.9)
Abscess drainage	69 (10.2)	12 [7–17]	
Open-abdomen revision	50 (7.4)	1 [1–3]	
Need of reoperation	129 (19.1)	4 [2–9]	
Medical complications	361 (53.6)	1 [1–2]	
Respiratory dysfunction	239 (35.5)	1 [1–2]	14 (5.9)
Pneumonia	190 (28.2)	1 [1–3]	27 (14.2)
Pulmonary embolism	26 (3.9)	3 [1–7]	10 (38.5)
TIA	9 (1.3)	14 [1–20]	5 (55.6)
Kidney dysfunction	113 (16.8)	1 [1–1]	4 (3.5)
Liver dysfunction	30 (4.5)	1 [1–1]	2 (6.7)
Asystole and resuscitation	9 (1.3)	3 [1–5]	3 (33.3)
Heart failure	11 (1.6)	1 [1–5]	4 (36.4)
AF	33 (5.0)	1 [1–2]	1 (3.0)
Septicemia	164 (24.3)		
Need of hemodynamic support	164 (24.3)		
High-output stoma	17 (2.5)		
Postoperative ileus	123 (18.2)		
Diarrhea	11 (1.6)		

TIA transient ischemic attack; *AF* atrial fibrillation

**Table 2:** Types of complications according to the Clavien-Dindo classification in 674 patients after emergency laparotomy

Clavien-Dindo classification	
I	41 (10.5)
II	86 (22.1)
IIIa	24 (6.2)
IIIb	35 (9.0)
IVa	54 (13.9)
IVb	107 (27.5)
V	42 (10.8)

The patients with operation-related complications had higher ASA class, a higher rate of excessive alcohol consumption and a longer duration of the operation compared with the patients with no operation-related complications. The patients whose operation was contaminated or those with stoma had a higher rate of operation-related complications.

ICU admissions were more common in the patients with operation-related complications compared with those without. Hospital LOS and ICU LOS were longer in patients with operation-related complications. Compared with the patients with no operation-related complications, both hospital and 90-day mortalities were higher in the patients with operation related complications (Table 3).

**Table 3:** Comparison of 674 patients with and without operation-related complications after emergency laparotomy

	No operation-related complication *N* = 459	Operation-related complication *N* = 215	*p *value
Age	67 [56–77]	66 [54–74]	0.079
Sex (male)	248 (54.0)	123 (57.2)	0.439
ASA class	3 [2–4]	3 [3–4]	< 0.001
Operation time (min)	90 [59–130]	114 [77–157]	< 0.001
Preoperative BMI	25.3 [22.2–28.6]	26.1 [23.2–29.7]	0.066
Smoker	63 (13.7)	37 (17.2)	0.236
Illicit drug abuse	7 (1.5)	8 (3.7)	0.072
Excessive alcohol consumption	39 (8.5)	36 (16.7)	0.002
*Urgency*			
Immediate (operation within 3 h)	202 (44.0)	135 (62.8)	< 0.001
Very urgent (operation within 3–8 h)	135 (29.4)	47 (21.9)	0.040
Urgent (operation within 8–24 h)	122 (26.6)	33 (15.3)	0.001
*History of abdominal surgery*			
Previous abdominal surgery	180 (39.2)	75 (34.9)	0.280
Reoperation during the same hospital stay	48 (10.5)	43 (20.0)	0.001
Malignancy	162 (35.3)	81 (37.7)	0.549
CCI	4 [2–6]	4 [2–7]	0.413
No chronic comorbidities	81 (17.6)	31 (14.4)	0.294
*Operation diagnosis*			
Malignancy/tumor	48 (10.5)	14 (6.5)	0.099
GI ulcer	39 (8.5)	11 (5.1)	0.119
Hernia	38 (8.3)	10 (4.7)	0.088
Diverticulitis/colitis	30 (6.5)	11 (5.1)	0.472
Ileus/occlusion	141 (30.7)	35 (16.3)	< 0.001
Peritonitis	9 (2.0)	11 (5.1)	0.024
Vascular cause	32 (7.0)	19 (8.8)	0.393
HBP	2 (0.4)	10 (4.7)	< 0.001
Other GI diseases	51 (11.1)	32 (14.9)	0.165
Injury	10 (2.2)	10 (4.7)	0.078
Other rare causes	12 (2.6)	3 (1.4)	0.317
Postoperative complication	47 (10.2)	49 (22.8)	< 0.001
*Operation type*			
Abdominal wall, mesentery, peritoneum and greater omentum	192 (41.8)	91 (42.3)	0.903
Upper GI tract	37 (8.1)	13 (6.0)	0.352
Small intestine and colorectal surgery	214 (46.6)	93 (43.3)	0.413
HBP	0	3 (1.4)	0.011
GI complication	15 (3.3)	15 (7.0)	0.030
Contaminated operation	134 (29.2)	109 (50.7)	< 0.001
Colon or small intestine resection	195/42.5)	105 (48.8)	0.122
Stoma	96 (20.9)	70 (32.6)	0.001
Primary suturing or anastomosis	204 (44.4)	79 (36.7)	0.059
CRP (mg/l), preop	39 [7–147]	126 [25–248]	< 0.001
Hemoglobin (g/l), preop	126 [109–141]	117 [98–133]	< 0.001
Leukocytes (E9/l), preop	10 [6.9–13.6]	11.5 [7.6–16.3]	0.010
Albumin (g/l), preop *	29 [24–33]	24 [20–29]	< 0.001
Creatinine (umol/l), preop	71 [55–96]	77 [56–123]	0.049
Hospital LOS (days)	8 [5–11]	21 [12–34]	< 0.001
Preoperative LOS (days)	1 [0–2]	1 [0–4]	0.036
Postoperative LOS (days)	6 [4–9]	18 [9–29]	< 0.001
ICU admission	94 (20.5)	123 (57.2)	< 0.001
ICU LOS	3 [2–5]	8 [4–17]	< 0.001
Hospital mortality	34 (7.4)	27 (12.6)	0.030
30-day mortality	48 (10.5)	29 (13.5)	0.249
90-day mortality	68 (14.8)	52 (24.2)	0.003
*Discharge location*			
Home	227 (49.5)	70 (32.6)	< 0.001
Health center ward	173 (37.7)	92 (42.8)	0.206
Central hospital	20 (4.4)	24 (11.2)	0.001
Residential/nursing home	4 (0.9)	2 (0.9)	0.940
Prison hospital	1 (0.2)	0	0.493

*n*, (%) or median, [25th to 75th percentile]

*ASA* American Society of Anesthesiologists, *BMI* body mass index, *CCI* Charlson Comorbidity Index, *GI* gastrointestinal, *HBP* hepatopancreaticobiliary, *CRP* C-reactive protein, *LOS* length of stay, *ICU* intensive care unit

^*^Missing data *n* = 221/95

The patients with medical complications were older and had higher ASA class and CCI scores, a longer duration of operation and a higher rate of very urgent operations compared with the patients without medical complications. The patients with EL due to ulcer or hernia had a lower rate of medical complications compared with the patients with other causes of EL. The patients whose emergency laparotomy was contaminated or those with stoma had a higher rate of medical complications. Primary suturing and anastomosis were more common among patients without medical complications (Table 4).

**Table 4:** Comparison of 674 patients with or without medical complications after emergency laparotomy

	No medical complication *N* = 313	Medical complication *N* = 361	*p* value
Age	64 [50–75]	68 [59–78]	< 0.001
Sex (male)	156 (49.8)	215 (59.6)	0.011
ASA class	3 [2–3]	3 [3–4]	< 0.001
Operation time (min)	85 [59–130]	106 [71–149]	< 0.001
Preoperative BMI	25.4 [22.3–29.2]	25.6 [22.4–28.7]	0.626
Smoker	38 (12.1)	62 (17.2)	0.067
Illicit drug abuse	8 (2.6)	7 (1.9)	0.588
Excessive alcohol consumption	28 (8.9)	47 (13.0)	0.093
Urgency			
Immediate (operation within 3 h)	141 (45.0)	196 (54.3)	0.017
Very urgent (operation within 3–8 h)	83 (26.5)	99 (27.4)	0.792
Urgent (operation within 8–24 h)	89 (28.4)	66 (18.3)	0.002
History of abdominal surgery			
Previous abdominal surgery	118 (37.7)	137 (38.0)	0.947
Reoperation during the same hospital stay	35 (11.2)	56 (15.5)	0.101
Malignancy	102 (31.7)	141 (40.1)	0.081
CCI	3 [1–6]	5 [3–7]	< 0.001
No chronic comorbidities	67 (21.4)	45 (12.5)	0.002
Operation diagnosis			
Malignancy/tumor	28 (8.9)	34 (9.4)	0.832
GI ulcer	32 (10.2)	18 (5.0)	0.010
Hernia	34 (10.9)	14 (3.9)	< 0.001
Diverticulitis/colitis	24 (7.7)	17 (4.7)	0.109
Peritonitis	5 (1.6)	15 (4.2)	0.051
Ileus/occlusion	83 (26.5)	93 (25.8)	0.824
Vascular cause	18 (5.8)	33 (9.1)	0.097
HBP	3 (1.0)	9 (2.5)	0.133
Other GI diseases	14 (4.5)	21 (5.8)	0.433
Injury	9 (2.9)	11 (3.0)	0.896
Other rare causes	9 (2.9)	6 (1.7)	0.287
Postoperative complication	36 (11.5)	60 (16.6)	0.058
Operation type			
Abdominal wall, mesentery, peritoneum and greater omentum	133 (42.5)	150 (41.6)	0.805
Upper GI tract	30 (9.6)	20 (5.5)	0.046
Small intestine and colorectal surgery	135 (43.1)	172 (47.6)	0.241
HBP	0	3 (0.8)	0.106
GI complication	15 (4.8)	16 (4.4)	0.824
Contaminated operation	98 (31.3)	145 (40.2)	0.017
Colon or small intestine resection	135 (43.1)	165 (45.7)	0.502
Stoma	62 (19.8)	104 (28.8)	0.007
Primary suturing or anastamosis	145 (46.3)	138 (38.2)	0.034
CRP (mg/l), preop	35 [5–133]	94 [21–218]	< 0.001
Hemoglobin (g/l), preop	127 [113–142]	117 [100–135]	< 0.001
Leukocytes (E9/l), preop	9.7 [7.1–13.5]	11.1 [7.4–15.3]	0.134
Albumin (g/l), preop*	29 [25–33]	26 [22–31]	0.001
Creatinine (umol/l), preop	68 [55–88]	79 [58–121]	< 0.001
Hospital LOS (days)	7 [5–11]	14 [8–25]	< 0.001
Preoperative LOS (days)	1 [0–3]	1 [0–3]	0.094
Postoperative LOS (days)	6 [4–8]	12 [7–22]	< 0.001
ICU admission	30 (9.6)	187 (51.8)	< 0.001
ICU LOS	2 [1–4]	5 [3–12]	< 0.001
Hospital mortality	11 (3.5)	50 (13.9)	< 0.001
30-day mortality	15 (4.8)	62 (17.2)	< 0.001
90-day mortality	25 (8.0)	95 (26.2)	< 0.001
Discharge location			
Home	196 (62.6)	101 (28.0)	< 0.001
Health center ward	90 (28.8)	175 (48.5)	< 0.001
Central hospital	13 (4.2)	31 (8.6)	0.020
Residential/nursing home	2 (0.6)	4 (1.1)	0.518
Prison hospital	1 (0.3)	0	0.283

*n* (%) or median [25th–75th percentile]

*ASA* American Society of Anesthesiologists, *BMI* body mass index; CCI, Charlson Comorbidity Index; *GI* gastrointestinal; *HBP* hepatopancreaticobiliary; *CRP* C-reactive protein; *LOS*, length of stay; *ICU* intensive care unit

^*^Missing data *n* = 160/156

ICU admissions were more common in patients with medical complications, and ICU LOS and hospital LOS were significantly longer compared with the patients without medical complications. The hospital, 30- and 90-day mortalities were significantly higher in the patients with medical complications compared with the patients without medical complications (Table 4).

The hospital, 30- and 90-day mortalities were higher among patients with medical complications (13.9%, 17.2%, 26.2%) compared with the operation-related complication group (12.6%, 13.5%, 24.2%). ICU admissions were more common, and ICU LOSs were longer in the operation-related complication group (57.2%, 8 [4–17] days) compared with the medical complication group (51.8%, 5 [3–12] days). (Tables 3 and 4).

### Multivariate analysis

According to the multivariate analysis, lower albumin level, higher surgical urgency, excessive alcohol consumption, higher preoperative body mass index (BMI) and medical complications were associated with operation-related complications (Table 5). Older age, higher ASA class and operation-related complications were associated with medical complications (Table 6.)

**Table 5:** Variables associated with operation-related complications in 674 patients after emergency laparotomy analyzed with logistic regression model

Factor	OR (95% Cl)	*p* value
Albumin, preoperative	0.95 (0.92–1.00)	0.016
Immediate surgery (operation within 3 h)	1	
Very urgent surgery (operation within 3–8 h)	0.35 (0.18–0.70)	0.003
Urgent surgery (operation within 8–24 h)	0.43 (0.22–0.85)	0.015
Excessive alcohol consumption	2.48 (1.00–6.11)	0.049
Preoperative BMI	1.06 (1.01–1.11)	0.020
Medical complication	6.74 (3.57–12.74)	< 0.001

*BMI* body mass index

**Table 6:** Variables associated with medical complications in 674 patients after emergency laparotomy analyzed with logistic regression model

Factor	OR (95% Cl)	*p* value
Age	1.02 (1.01–1.04)	0.013
ASA class	1.78 (1.27–2.51)	0.001
CRP, preoperative	1.00 (1.00–1.01)	0.018
Operation related complication	7.02 (3.71–13.28)	< 0.001

*ASA* American Society of Anesthesiologists, *CRP* C-reactive protein

## Discussion

This study demonstrates that patients undergoing EL have a significant risk for both medical and operation-related complications. More than a half of the EL patients had at least one complication. The majority of the complications (50%) were medical complications and 30% were operation related. The medical complications were associated with patient-related factors, and operation-related complications were more often disease related. Medical complications occurred at an earlier phase of the postoperative care compared with operation-related complications.

In the present study, medical complications were recorded more often during the first 4 days after surgery, and operation-related complications developed at a later phase of recovery. Two thirds of the recorded complications were Clavien-Dindo class III or higher. Frailty, high ASA class, low albumin level and age have been reported as risk factors for complications [33]. Also in this study, age, high ASA class and higher preoperative CRP were associated with medical complications.

Medical complications were recorded earlier compared to operation-related complications, which may indicate that patients' deterioration due to medical complications may increase the risk for operation-related complications. It has been shown that pulmonary diseases increase the risk for anastomotic leakage after colon surgery [34], and wound infections are associated with fascial rupture and abdominal wall dehiscence [35]. As in these elective settings, also in this study medical complication may increase the risk for operation-related complication; however, causation could not be demonstrated.

According to our knowledge, the present study is the first one focusing on the onset of complications. Systemic dysfunction caused by inflammation is already present before surgery in the EL patients, whereas for the elective procedures the systematic inflammatory response is more often a postoperative phenomenon [36]. Similar factors associated with complications were found in a previous study focusing on elective patients undergoing free flap surgery for cancer of the head and neck [32]. In this elective setting, both complication types occurred more often late than early [32]. Although the patient cohort in that previous study was significantly different compared with ours, the factors associated with complications were similar.

The patients requiring an urgent surgery have several predisposing factors for systemic inflammatory response syndrome (SIRS), and the upregulation of the systemic inflammatory response is the primary cause of death in patients undergoing emergency surgery [36]. Already prior to laparotomy 30–50% of patients present SIRS, sepsis or septic shock [2–9]. This is also considered in ERAS protocols that are designed to minimize the physiological impact and stress response to surgical insult but in EL patients this insult and stress are already occurring prior to surgery [29]. These patients are also prone to infections because tissue injury following surgery or trauma increase the risk for infections [37]. In a Danish study, the median time from surgery to any surgical site infection or pulmonary complication was 9 days [38]. Difference in the time of onset of complications could be explained by the different of SIRS after EL.

The rate of complications in the present study is in line with previous studies [1, 39]. Wound infection was the most common operation-related complication as has been reported before [1]. However, we reported a higher rate of pneumonia compared with previous studies [3, 12].

Thirty- and 90-day mortality rates were high after EL in both medical and operation-related complication groups, and rates were similar compared to the previously reported rate [1, 4, 12, 40]. In this study the medical complications seemed to worsen the prognosis of the patient more than the operation-related complications.

Patients with old age and multiple comorbidities have a high risk for complications significantly impairing their prognosis. A British study found that age alone increases the 30-day mortality after EL [14]. In this study, patient-related factors were found to be significant for both medical and operation-related complications.

Since the intervention options to enhance the outcome are limited, EL is a high-risk operation regardless of the nature of the procedure. In elective abdominal surgery, ERAS protocols are commonly used to reduce complications and improve outcome [41]. Recent meta-analysis showed that ERAS reduced postoperative complications and hospital stay also in emergency surgery [42]. Although complete optimization of preoperative ERAS components is not achievable, the rest of the intra- and postoperative components of ERAS protocols are applicable and appropriate also in the emergency setting [42]. Information about the incidence of complications after emergency laparotomy could help to develop the ERAS protocols further and also improve active rehabilitation immediately after surgery. During this study the ERAS protocol was not applied to the EL patients in our hospital.

Some previous studies have demonstrated that emergency laparoscopic surgery is associated with a lower rate of complications compared with the emergency laparotomy [2, 10]. Even though laparoscopy has become increasingly common nowadays, it cannot fully replace the EL, and the rate of procedure conversion to open laparotomy remains high [43]. The most complex cases, unstable patients and those with several previous laparotomies are not suitable for laparoscopy. In some cases, the length of operation may create a problem since longer duration of surgery is associated with postoperative complications [44]. This highlights the importance of meticulous patient selection when choosing the right method for emergency surgery.

The risk of complications is high after EL, and therefore consideration is required in the treatment of these patients. At least in some high-risk patient groups palliative care could be an option as surgery is unlikely to extend the patient's life expectancy in a positive manner. There are a few studies focusing on the palliative care as an option for surgery, but their results do not provide an unambiguous answer to the question of which patients should be referred to non-operative treatment [45, 46]. Hopefully further studies will provide more information on how to choose between operative or conservative treatment in frail patients in need of emergency laparotomy.

The surgical insult of a major operation like laparotomy increases the inflammatory response, which can predispose especially the frail and comorbid patients to medical complications [45, 46]. One could hypothesize that these medical complications could be one factor predisposing patients later to surgical complications. However, the present setting does not allow us to show any causality between the medical and surgical complications.

## Limitations

This study has a few limitations. The present study is a retrospective single-center cohort study, which may impair the generalization of the results. However, the rate and types of complications were in line with the previous studies. We included all EL patients in our hospital during the study period; thus, the risk of selection bias is low. As a limitation we did not have access to data on the acute physiology of the patients. Due to the retrospective study design, some patient data were missing, and we are not able to demonstrate causality between medical and operation-related complications.

## Conclusion

Operation-related complications occurred later after surgery compared with medical complications. The type of EL did not have a significant impact on the rate of complications. Low albumin level, high preoperative CRP, older age, and high ASA class increase the risk for medical complications after EL.
